# Identification and ultrastructural characterization of *Acanthamoeba* bacterial endocytobionts belonging to the *Alphaproteobacteria* class

**DOI:** 10.1371/journal.pone.0204732

**Published:** 2018-10-24

**Authors:** Li Li Chan, Joon Wah Mak, Stephen Ambu, Pei Yee Chong

**Affiliations:** 1 Pathology Division, School of Medicine, International Medical University, Kuala Lumpur, Malaysia; 2 School of Postgraduate Studies and Research, International Medical University, Kuala Lumpur, Malaysia; 3 Medical Sciences, School of Medicine, International Medical University, Kuala Lumpur, Malaysia; Free University of Bozen/Bolzano, ITALY

## Abstract

The detection and identification of two endocytobiotic bacterial strains, one affiliated to the “*Candidatus* Caedibacter acanthamoebae”/“*Ca*. Paracaedimonas acanthamoeba”, and another to the endosymbiont of *Acanthamoeba* UWC8 and “*Ca*. Jidaibacter acanthamoeba” are described. For endocytobiont screening, we developed a PCR method with a set of broad-range bacterial 16S rRNA primers to substitute the commonly used but technically demanding fluorescent *in situ* hybridization technique. Our PCR test alone without sequencing failed to discriminate the endocytobiont-containing and endocytobiont-free *Acanthamoeba* sp. due to the presence of mismatched primers to host mitochondrial DNA. We highlighted the need to perform bacterial primer checking against the *Acanthamoeba* genome to avoid false positive detection in PCR. Although the genetic aspect of “*Ca*. Caedibacter acanthamoebae”/“*Ca*. Paracaedimonas acanthamoeba” and the endosymbiont of *Acanthamoeba* UWC8/“*Ca*. Jidaibacter acanthamoeba” are well studied, knowledge pertaining to their morphologies are quite vague. Hence, we used transmission electron microscopy to examine our endocytobionts which are affiliated to previously described intracellular bacteria of *Acanthamoeba* sp. We used good-quality TEM images for the localization and the fate of the current endocytobionts inside different life stages of the hosts. Furthermore, to the best of our knowledge, our TEM findings are the first to provide morphological evidence for the clearance of defective *Acanthamoeba* endocytobionts via an autophagic-like process.

## Introduction

*Acanthamoeba* species are free-living amoebae which are widely distributed in the natural and built environments [1–2]. The microorganism has two stages in its life cycle, a dormant cystic stage and a vegetative trophic stage. The cyst has double-layered walls which protect it from unfavorable growth conditions [1]. Cyst formation also facilitates the acanthamoebal persistence and ubiquitous dissemination in the environment [1–2]. The trophozoite is the metabolically active stage which obtains its energy by consuming bacteria, yeast, small protists and organic particles [3]. Certain *Acanthamoeba* sp. are amphizoic parasites which infect humans and cause neurological, ocular and cutaneous diseases [1–2]. A number of parasitic factors have been associated with the virulence potential of the pathogenic strains [4]. Perhaps, more parasite virulence factors will be discovered in near future.

Historically, *Acanthamoeba* species were classified into three morphological groups (Group I, II and III) based on the size and shape of cysts [5]. Nevertheless, the morphological-based classification can be ambiguous due to inconsistent cyst features. The current trend in *Acanthamoeba* taxonomy favours the use of molecular genotyping, in which the *Acanthamoeba* genus has been classified into 17 genotypes (T1–T17) based on the 18S rRNA gene sequences [6–11]. T4 is the most abundant *Acanthamoeba* genotype isolated from the environment; it is also the most predominant genotype associated with human infections [12–14].

In recent years, there has been growing interest in investigating the roles of *Acanthamoeba* as hosts, reservoirs and vectors for endocytobionts [15–17]. The term ‘endocytobionts’ refers to bacteria, fungi, small protozoa or viruses which are able to reside permanently or transiently in the cellular milieu of the amoebae [15–16, 18]. The interaction between an endocytobiont and its host is either mutualistic, commensalistic or parasitic, and the relationship can be altered by external factors such as the growth temperature of the host [15, 17]. At present, an abundant list of endocytobionts have been identified in clinical and environmental isolates of *Acanthamoeba* sp. [15–18]. While the impact of most endocytobionts on the wellbeing of humans is yet to be determined, a number of the naturally occurring endocytobiotic bacteria and viruses have been identified as human pathogens or potential pathogens [19–22]. The endocystic wall of *Acanthamoeba* are cellulose-rich, making the cyst an ideal stage which protects *Acanthamoeba* as well as their endocytobionts from external toxins including the microbiocidal molecules of our immune cells [16, 23–25]. Hence, it is believed that the harmful or potentially harmful endocytobionts may exploit *Acanthamoeba* cyst as a Trojan horse to enter the host. In addition, several studies have shown that the phagocytosis and digestion mechanisms of *Acanthamoeba* and macrophages shared common features, suggesting microbes which could survive predation by an *Acanthamoeba* could also survive predation by a macrophage [16, 26].

In a previous study to determine the occurrence of *Acanthamoeba* sp. in air-conditioning units, we isolated and established twenty-one clonal isolates of *Acanthamoeba* [27]. We carried out axenization procedure to eliminate bacterial contaminants, grew the amoebae in peptone-yeast-glucose (PYG) medium and studied their morphological, genotypic and physiological characteristics [27]. Even with proper aseptic culture procedures, we frequently observed bacterial-like structures in the medium nourishing five of the environmental *Acanthamoeba* isolates and one newly established keratitis isolate. Attempts to isolate these bacteria on blood agar or nutrient agar were unsuccessful. This led us to suspect that they originated from bacterial endocytobionts in these amoebae isolates.

In this study, we opted to develop a PCR method with a set of broad-range primer pairs which target the bacterial 16S rRNA genes to substitute the commonly used but technical demanding fluorescent *in situ* hybridization (FISH) technique for the screening of bacterial endocytobionts in our *Acanthamoeba* isolates [28–29]. Subsequently, we performed nucleotide sequence analysis on the amplified gene sequences and identified two bacterial endocytobiont strains, one affiliated to the previously described “*Candidatus* Caedibacter acanthamoebae” and another to the endosymbiont of *Acanthamoeba* UWC8/“*Ca*. Jidaibacter acanthamoeba” [8, 30]. Recently, the taxonomy of “*Ca*. Caedibacter acanthamoebae” has been revised; the bacteria was proposed to be renamed as “*Ca*. Paracaedimonas acanthamoeba” [31]. These previously described intracellular bacteria belong to the *Alphaproteobacteria* class and their genome have been extensively studied (Genbank accession no. CP008936.1, NZ_CP004403.1 and NZ_JSWE01000000); on the contrary, knowledge pertaining to their morphologies remains quite limited [8, 30–35]. Of importance, to the best of our knowledge, ultrastructural information revealing the endocytobiont localizations and their fate inside different life stages of naturally infected *Acanthamoeba* host cells remain unexplored. Hence, in this study, we further examined the ultrastructural features our *Acanthamoeba* isolates bearing the two endocytobionts which are affiliated to the “*Ca*. Caedibacter acanthamoebae”/“*Ca*. Paracaedimonas acanthamoeba” and the endosymbiont of *Acanthamoeba* UWC8/“*Ca*. Jidaibacter acanthamoeba”, respectively, by transmission electron microscopy (TEM). In conjunction to nucleotide sequence analysis, we performed FISH assay with oligonucleotide probes which are specific to “*Ca*. Caedibacter acanthamoebae”/“*Ca*. Paracaedimonas acanthamoeba” and the endosymbiont of *Acanthamoeba* UWC8/“*Ca*. Jidaibacter acanthamoeba”, respectively, to confirm the intracellular localization of our endocytobionts prior to TEM study [8, 30]. Our TEM findings have successfully provided a richer perspective on host-endocytobiont interactions. In addition, our TEM observations suggest hints for new features of interaction, such as the involvement of autophagy in removing defective endocytobiont by its host.

## Materials and methods

### *Acanthamoeba* cultures

Eleven isolates from air-conditioners (IMU4, IMU5, IMU7, IMU8, IMU9, IMU11, IMU12, IMU13, IMU14, IMU17 and IMU19) and two keratitis isolates (HTH136 and HKL55, isolated from Malaysian patients) of *Acanthamoeba* sp. were included in this study [27]. Except IMU14 and IMU17 which is a T3 and T5 isolate respectively, all other *Acanthamoeba* isolates belong to the T4 genotype [27]. A keratitis isolate of *A*. *castellanii* (strain CDC:0184:V014; ATCC 50492) was used as positive control for PCR. All isolates were maintained in PYG medium at ambient temperature (~26°C ± 2) following the protocol as described elsewhere [36].

### DNA extraction, PCR, amplicon purification, sequencing and sequence analysis

*Acanthamoeba* trophozoites were harvested from PYG cultures, washed twice with 1x PBS (OmniPur, Merck, Germany) and resuspended in 200 μl PBS. DNA was extracted from the cells using the QIAamp DNA mini kit (Qiagen, Hilden, Germany). PCR detection of endocytobiotic bacteria was performed using a set of universal primers for bacterial 16S rRNA gene (331F, 5′-TCCTACGGGAGGCAGCAGT-3′; 797R, 5′-GGACTACCAGGGTATCTAATCCTGTT-3′) which amplified ~400 bp of the gene segment [29]. In order to amplify a longer (~940 bp) segment of the 16S rRNA gene, another set of universal bacterial 16S rRNA gene primers (UnF, 5’-GAGTTTGATCCTGGCTCAG-3’; E939R, 5’-CTTGTGCGGGCCCCCGTCAATTC-3’) were used in PCR [37–38]. PCR mixtures were set up with the TopTaq Master Mix (Qiagen, Hilden, Germany) according to the manufacturer’s protocol, with each primers and DNA templates adjusted to 0.5 μM and ~0.5 μg in 50 μl reaction, respectively. PCR with primers 331F-797R was carried out with 20 cycles of thermal program: denaturation, 94°C for 1 min; annealing, 56°C for 30 s; and elongation, 72°C for 30 s. As for the primers UnF-E939R, PCR was carried out with 30 cycles of thermal program: denaturation, 94°C for 1 min; annealing, 52°C for 1 min; and elongation, 72°C for 1 min. All amplicons were purified using the QIAquick gel extraction kit (Qiagen, Hilden, Germany) and sequenced directly at both strands using the amplification primers in an Applied Biosystems 3100 sequencer (First BASE Laboratories, Malaysia). The sequences determined were aligned using the Clustal Omega software whereas nucleotide sequence analysis was performed using the nucleotide BLAST (Blastn) program of the National Center for Biotechnology Information and the Needle global sequence alignment program of the European Molecular Biology Open Software Suite [39–41]. DNA sequences determined in this study have been deposited in the Genbank database under accession numbers: KX257185–KX257198, MF688840–MF688843.

### Fluorescent *in situ* hybridization assay

Approximately 1 ml amoebae culture was transferred from tissue culture flask into a 1.5 ml-microcentrifuge tube. The cells were washed once with PBS and fixed with 4% formaldehyde solution, buffered, pH 6.9 (Merck, Germany), for 20 mins. Approximately 2 μl fixed cells were spotted onto a poly-L-lysine (Sigma, USA) coated microscopic slide and the slide was air-dried at room temperature. The cells were dehydrated through an ethanol series (50%, 80% and 90%, 3 mins each) and air-dried at room temperature before subjected to FISH assay [42].

We performed double FISH with a probe which specifically hybridized to the complementary sequence of 16S rRNA of the targeted endocytobiont, together with a probe which hybridized to the 16S rRNA of most bacteria. Oligonucleotide probes specific for “*Ca*. Caedibacter acanthamoebae”/“*Ca*. Paracaedimonas acanthamoeba” (S-S-CaeAc-998-a-A-18) and for the endosymbiont of *Acanthamoeba* UWC8/“*Ca*. Jidaibacter acanthamoeba” (S-*-AnEnd-1196-a-A-18), respectively, were conjugated to fluorescein isothiocyanate (FITC) dye, whereas the bacterial-domain-specific probe (S-D-Bact-0338-a-A-18) was conjugated to sulfoindocyanine Cy3 fluorescent dye [8, 30, 43]. We used FISH protocols as suggested with slight modifications [8, 30, 43–44]. Hybridization step was carried out in the dark at 46°C for 1.5 hrs. Optimal hybridization stringency for each combination of probes was tested by changing percentage of formamide (serial increment of 5%, from 10% to 25%) in hybridization buffer lacking SDS. Post-hybridization washing step was carried out in the dark at 48°C for 20 mins. All slides were air-dried at room temperature and the FISH-stained cells were visualized with a fluorescence microscope (Eclipse Ni-U, Nikon, Japan).

### Transmission electron microscopy

*Acanthamoeba* cells were harvested from PYG medium, washed thrice with PBS and pelleted by centrifugation (200x*g*, 5 min). The cell pellet was fixed with 4% ice-cold glutaraldehyde, post-fixed with 1% osmium tetroxide and 2% uranyl acetate, dehydrated with increasing concentrations of ethanol, and embedded in epoxy resin. Toluidine-blue stained, semi-thin sections of resin was prepared for sample block selection. Ultra-thin sections of 90 nm thickness were trimmed from the cell blocks and the sections were stained with 4% uranyl acetate-lead citrate solution. Internal structures of the cells were examined using the LEO-Libra 120 transmission electron microscope (Carl Zeiss AG, Germany) at an accelerating voltage of 80 kV, or by the Hitachi FESEM SU8000 ultra-high resolution scanning electron microscope (Hitachi High Technologies America, Inc., USA) at an accelerating voltage of 30 kV.

### Co-culturing of *Acanthamoeba* with *Escherichia coli*

Cells of an *Acanthamoeba* isolate which has been confirmed free from endocytobiont by the upstream molecular method and microscopic examination were harvested from PYG medium and transferred onto a non-nutrient agar plate seeded with *E*. *coli*. The cells were incubated for three days at ambient temperature. At the end of incubation, trophozoites were scraped from the agar surface, pelleted down, fixed in 4% glutaraldehyde in 0.1 M cacodylate buffer, followed by processing for TEM and viewing as described above.

## Results

### Molecular detection and identification of *Acanthamoeba* endocytobiotic bacteria

DNA extracted from all *Acanthamoeba* isolates, including the control strain, produced the ~400 bp-positive bands when amplified using the primers 331F-797R. The nucleotide sequences analyzed with Blastn program revealed only amplicons generated from DNA samples of IMU7, IMU11, IMU12, IMU13, IMU19 and HTH136 to have the highest identities (99–100%) with bacterial 16S rRNA genes. However, those which were amplified from the DNA samples of IMU4, IMU5, IMU8, IMU9, IMU14, IMU17, HKL55 and ATCC 50492 showed closest identities of 99–100% to the sequences of various *Acanthamoeba* mitochondrial 16S rRNA genes. Multiple sequence alignment analysis subsequently segregated the current six bacterial sequences into two 16S rRNA gene sequence types. One sequence type was represented by five identical bacterial sequences which were derived from the DNA of IMU11, IMU12, IMU13, IMU19 and HTH136 whereas another sequence type was represented by a single sequence which was derived from the DNA of IMU7. Global nucleotide sequence alignment analysis on the former five identical bacterial gene sequences showed a perfect match with respect to the 16S rRNA gene sequence belonging to “*Ca*. Caedibacter acanthamoebae”/“*Ca*. Paracaedimonas acanthamoeba” (GenBank accession no. CP008936.1), whereas the amplicon of IMU7 showed 100.0% and 99.2% (0.3% gaps) match in reference to the 16S rRNA genes belonging to the bacterial endosymbiont of *Acanthamoeba* sp. UWC8 (GenBank accession no. NZ_CP004403.1) and “*Ca*. Jidaibacter acanthamoeba” (GenBank accession no. NZ_JSWE01000000), respectively. Based on the molecular analysis, we successfully showed the presence of bacterial endocytobionts in six *Acanthamoeba* isolates. Hereafter, we named these endocytobionts as Endo_IMU7, Endo_11, Endo_IMU12, Endo_IMU13, Endo_IMU19 and Endo_HTH136. Furthermore, we amplified ~850 bp (excluding primers) segments of the 16S rRNA genes belonging to the Endo_IMU7, Endo_IMU12, Endo_IMU19 and Endo_HTH136, and analysed the nucleotide sequences again. Our nucleotide sequence analysis results consistently indicated the amplified 16S rRNA gene sequence of Endo_IMU7 to have 100.0% and 99.4% (0.2% gaps) identical matching with the corresponding genes of the bacterial endosymbiont of *Acanthamoeba* sp. UWC8 and “*Ca*. Jidaibacter acanthamoeba”, respectively, whereas that of the later three endocytobionts to have the highest identities (99.9% identity and 0.0% gap for Endo_HTH136; 100.0% identities for Endo_IMU12 and Endo_IMU19) with the “*Ca*. Caedibacter acanthamoebae”/“*Ca*. Paracaedimonas acanthamoeba” 16S rRNA gene sequence.

### Detection and intracellular localization of *Acanthamoeba* endocytobionts by FISH

**Fig 1:**
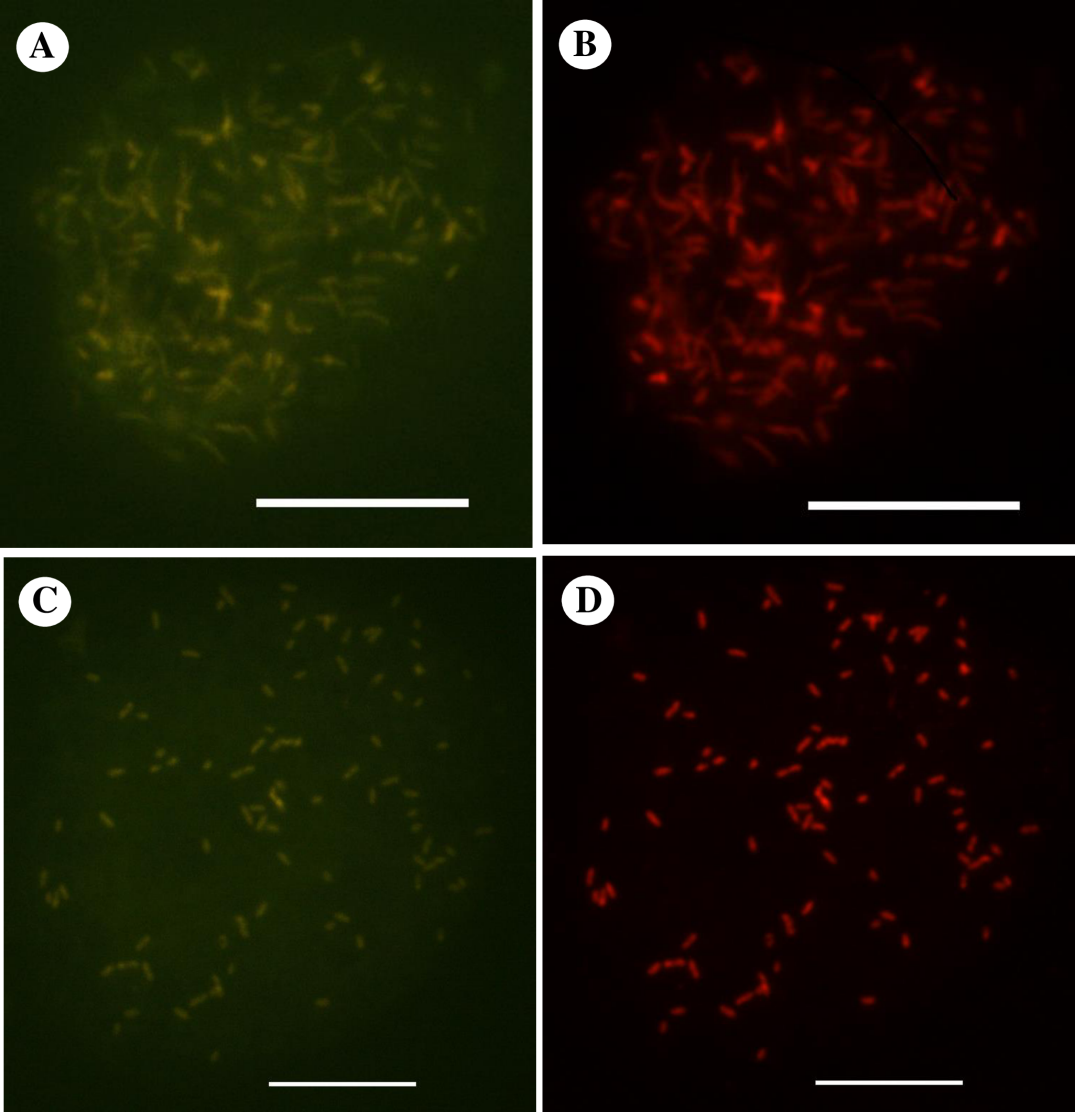
Representative double FISH images depicting the intracellular localization of bacterial endocytobionts investigated in this study. FISH detection of endocytobiont of *Acanthamoeba* sp. HTH136 (Endo_HTH136) by: (A) FITC-labelled probe specific to “*Ca*. Caedibacter acanthamoebae”/“*Ca*. Paracaedimonas acanthamoeba”, and (B) Cy3-labelled oligonucleotide bacterial-domain specific probe S-D-Bact-0338-a-A-18. FISH detection of endocytobiont of *Acanthamoeba* sp. IMU7 (Endo_IMU7) by: (C) FITC-labelled probe specific to the endosymbiont of *Acanthamoeba* UWC8/“*Ca*. Jidaibacter acanthamoeba”, and (D) Cy3-labelled S-D-Bact-0338-a-A-18 probe. For each combination of probes, an identical microscopic field was visualized by a fluorescence microscope. Bars represent 10 μm.

We used published endosymbiont-specific probes to detect and localize the current endocytobionts which were detected by the molecular method. Positive FISH signal was obtained with the “*Ca*. Caedibacter acanthamoebae”/“*Ca*. Paracaedimonas acanthamoeba” specific-probe for the endocytobionts of *Acanthamoeba* sp. HTH136 (Fig 1), IMU12 and IMU19 (S1 Fig). Likewise, positive FISH signal was spotted with the endosymbiont of *Acanthamoeba* UWC8/“*Ca*. Jidaibacter acanthamoeba”-specific probe for the endocytobiont of *Acanthamoeba* sp. IMU7 (Fig 1). In addition, we observed the bacterial probe S-D-Bact-0338-a-A-18 hybridizing simultaneously to the bacterial cells which were hybridized by either of the endosymbiont probes in their respective double FISH assay (Fig 1 and S1 Fig). For both double FISH assays, signal intensities were almost similar for hybridization buffers which contained 10% to 25% formamide.

### Ultrastructure of bacterial endocytobionts within their amoebae hosts

**Fig 2:**
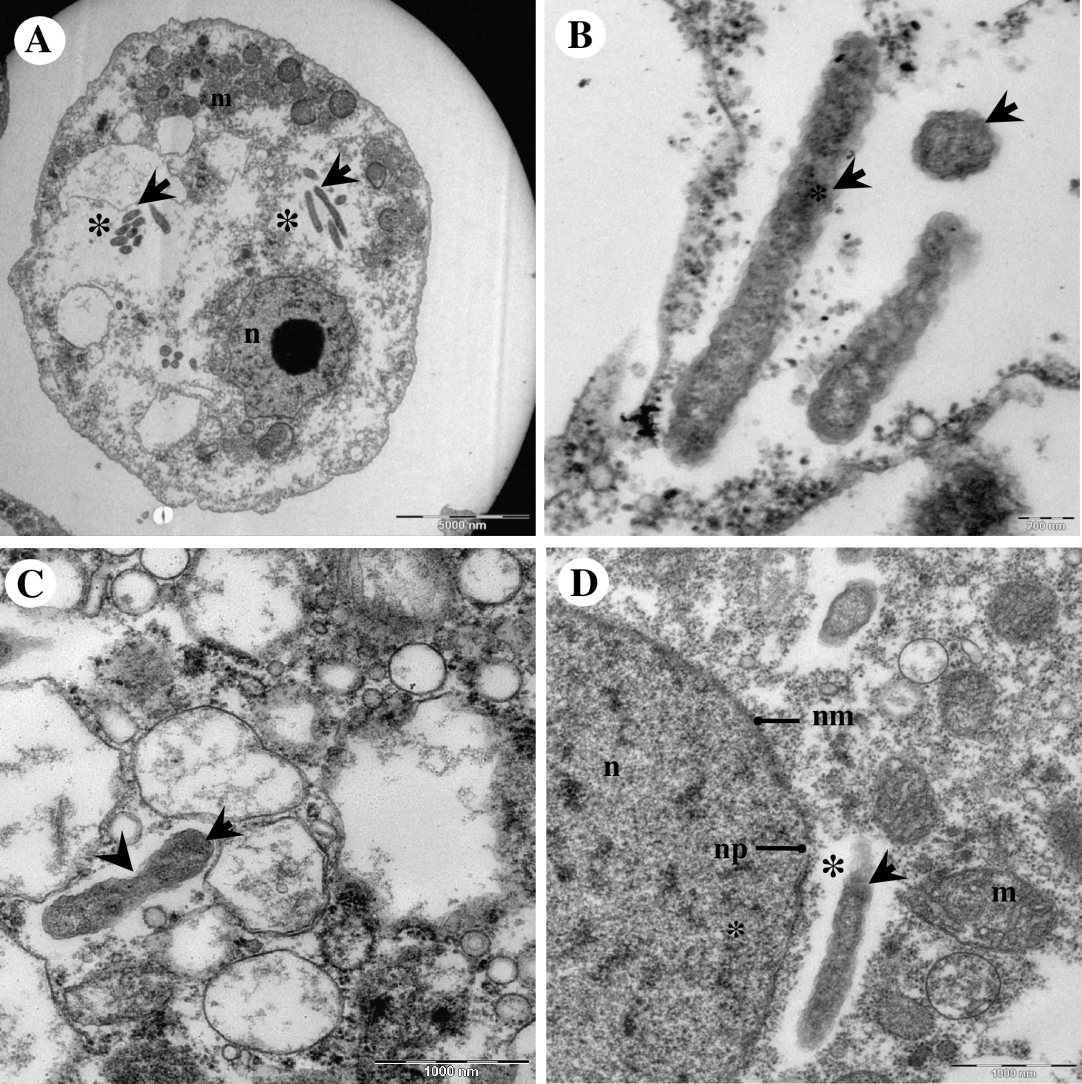
Transmission electron microscopy of *Acanthamoeba* sp. IMU12 and its endocytobiotic bacteria Endo_IMU12. (A) Overview of a trophozoite harbouring endocytobionts. (B) Higher magnification showing the pleomorphic, rod-shaped endocytobionts. (C) A bacterial cell undergoing binary fission. (D) A bacteria cell growing closely to host nuclear membrane. Indicators = endocytobionts: ‘arrows’, electron translucent space: ‘asterisks’, binary fission: ‘arrow-head’, mitochondria: m, nucleus: n, nuclear membrane: nm, and nuclear pore, np.

**Fig 3:**
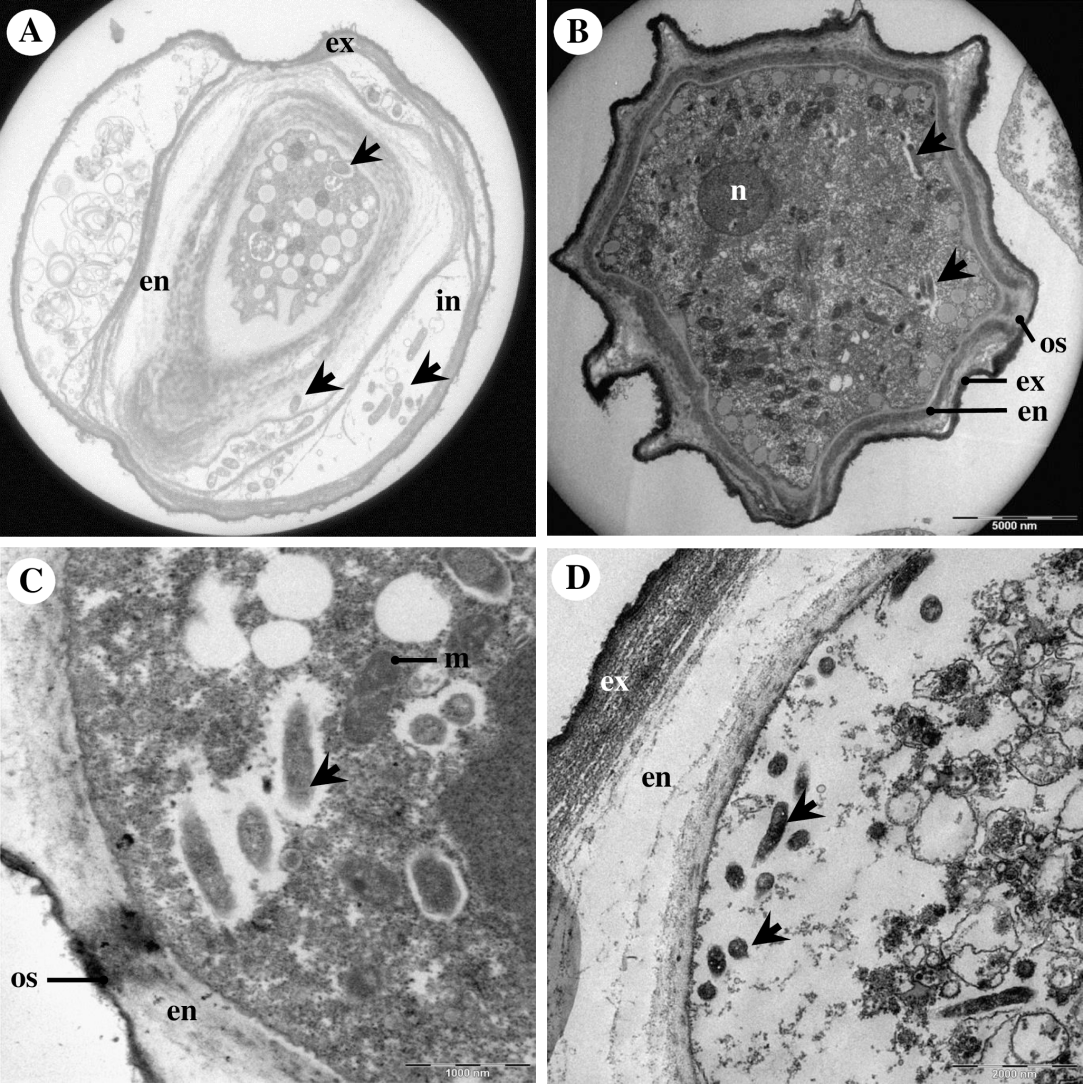
Ultrastructural features of endocytobionts in cystic stage of hosts. (A) Endo_HTH136 in *Acanthamoeba* sp. HTH136, (B) Endo_IMU12 in *Acanthamoeba* sp. IMU12, (C) Endo_IMU19 in *Acanthamoeba* sp. IMU19. (D). Several intact Endo_HTH136 are seen inside an empty cyst. Indicators: endocytobionts: ‘arrows’, endocyst: en, exocyst: ex, intercyst space: in, mitochondria: m, nucleus: n, and ostiole: os.

**Fig 4:**
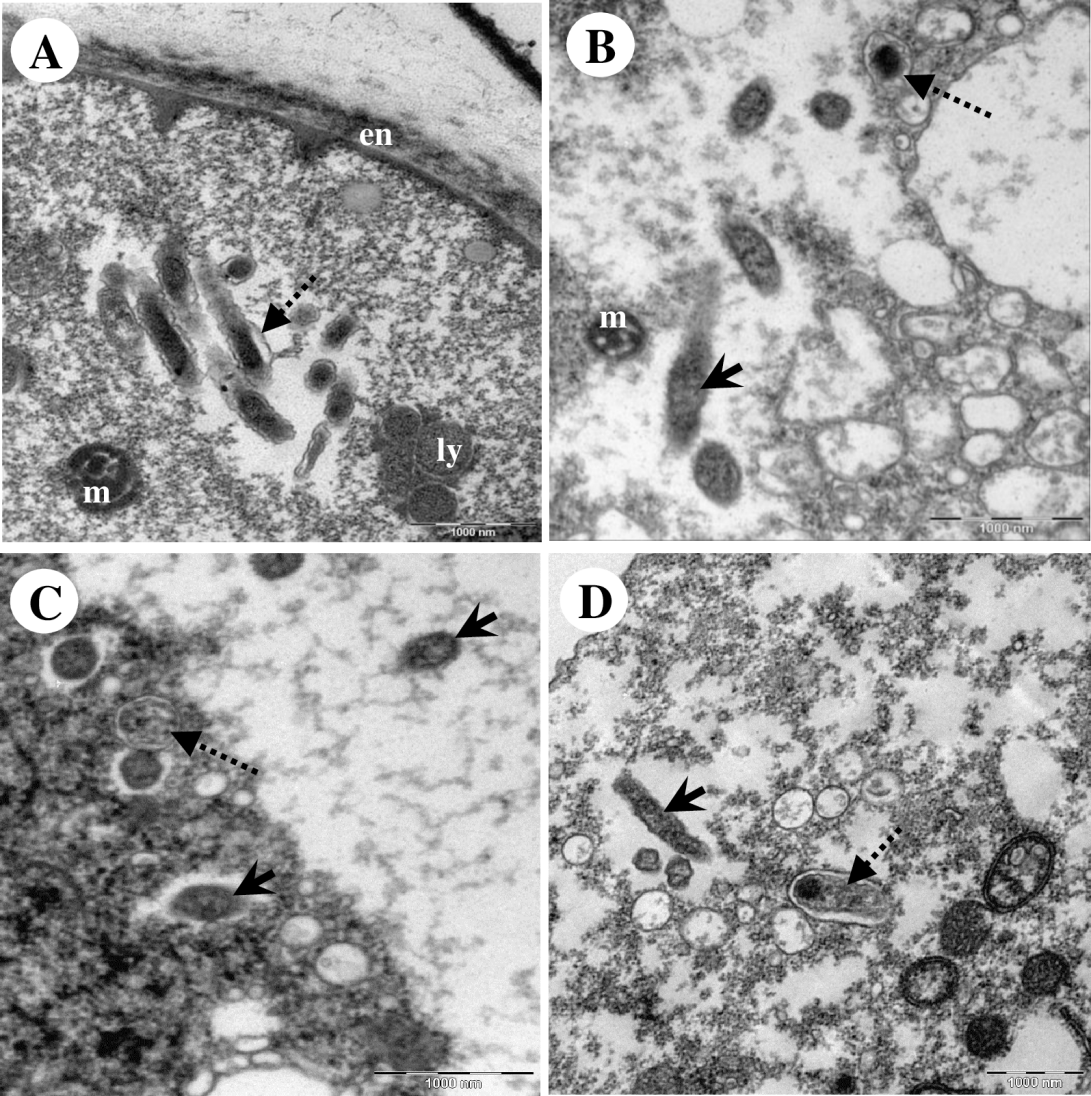
Transmission electron microscopy depicting the morphologically altered endocytobiots. (A) A cluster of morphologically altered Endo_IMU12 cells lie naked in the cytoplasm of a cyst. Morphologically altered (B) and (C) Endo_IMU19, and (D) Endo_HTH136 which were encircled by membranous structure. Note that only the morphologically altered but not the intact Endo_IMU19 and Endo_HTH136 were enclosed by membranous vacuole. Indicators: morphologically intact endocytobionts: ‘solid-arrows’, morphologically altered endocytobionts: ‘dotted-arrows’, endocyst: en, lysozyme: ly, and mitochondria: m.

By electron microscopy, Endo_IMU12, Endo_IMU19 and Endo_HTH136 appeared as pleomorphic rod-shaped bacteria which were non-membrane bound and were surrounded by electron-translucent regions of variable sizes (Fig 2). Most of the bacterial cells were distributed randomly throughout the cytoplasm of hosts. The cells multiplied by binary fission, occurred singly or in small clusters (Fig 2). No intranuclear stage was observed but a few cells which grew closely appended to nuclear membrane were spotted. (Fig 2). In cystic stage of hosts, Endo_HTH136 could be detected in the host’s cytoplasm, endocyst layer and intercyst space whereas for Endo_IMU12 and Endo_IMU19, they could only be detected in the cytoplasm of their respective hosts (Fig 3). Endo_IMU12, Endo_IMU19 and Endo_HTH136 cells which demonstrated altered morphologies, i.e. thickened and rough cell walls were infrequently observed within the cytoplasm of intact trophozoites and cysts (Fig 4). Uniquely, these cells were encircled by membranous structure and their cellular texture were either more or less electron-dense compared to the vast majority of cells (Fig 4). In contrast, morphologically intact Endo_IMU12, Endo_IMU19 and Endo_HTH136 cells could be seen in some empty or disintegrated cysts (Fig 3).

**Fig 5:**
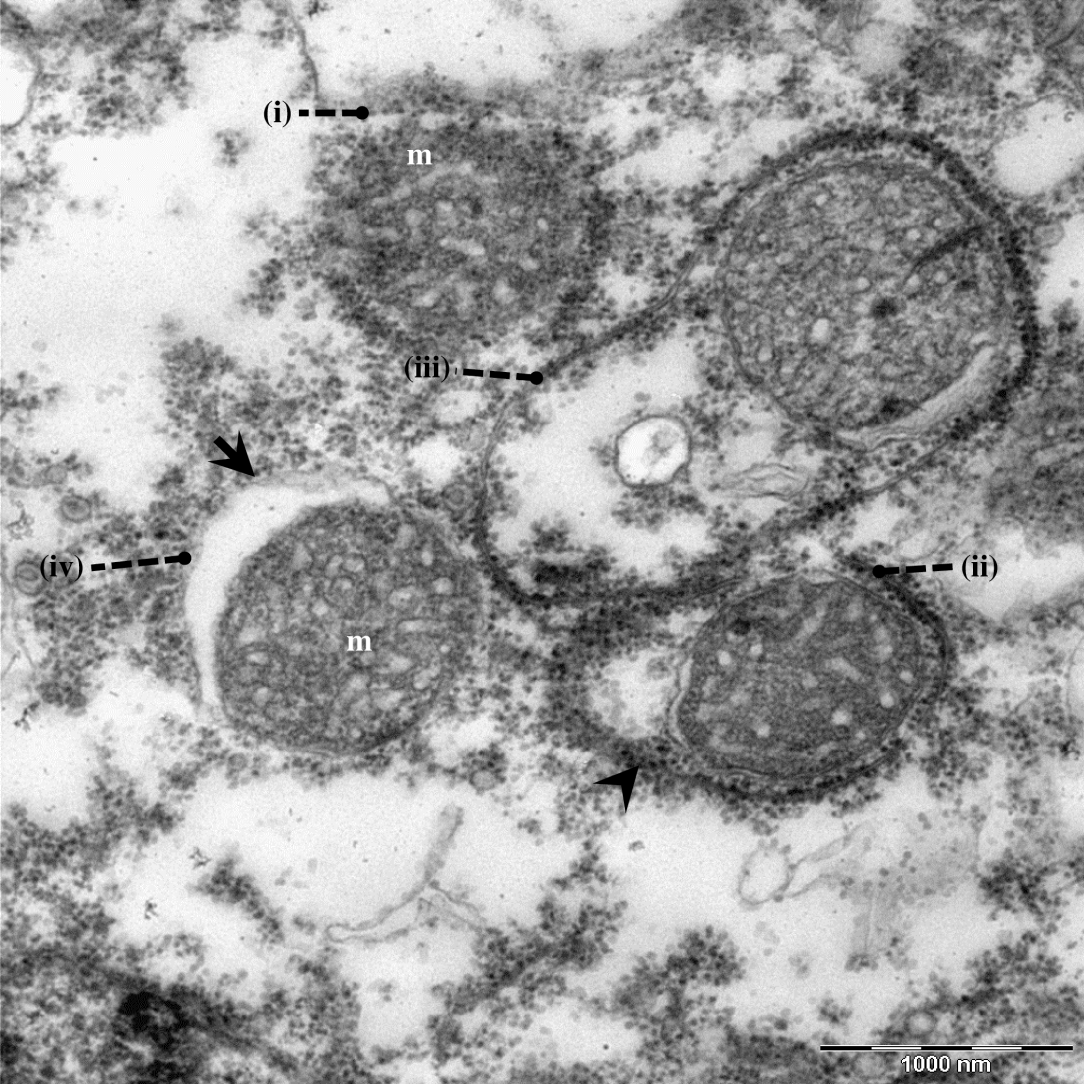
Transmission electron microscopic image illustrating autophagic removal of mitochondria in *Acanthamoeba* sp. (i) Autophagic isolation membrane appended to the targeted mitochondria, (ii) elongation of isolation membrane, (iii) elongated isolation membrane surrounding the entire mitochondria, (iv) a completely formed double-membranous autophagosome. Indicators = autophagic isolation membrane: ‘arrow-head’; autophagosomes: ‘arrow’, and mitochondria: m.

**Fig 6:**
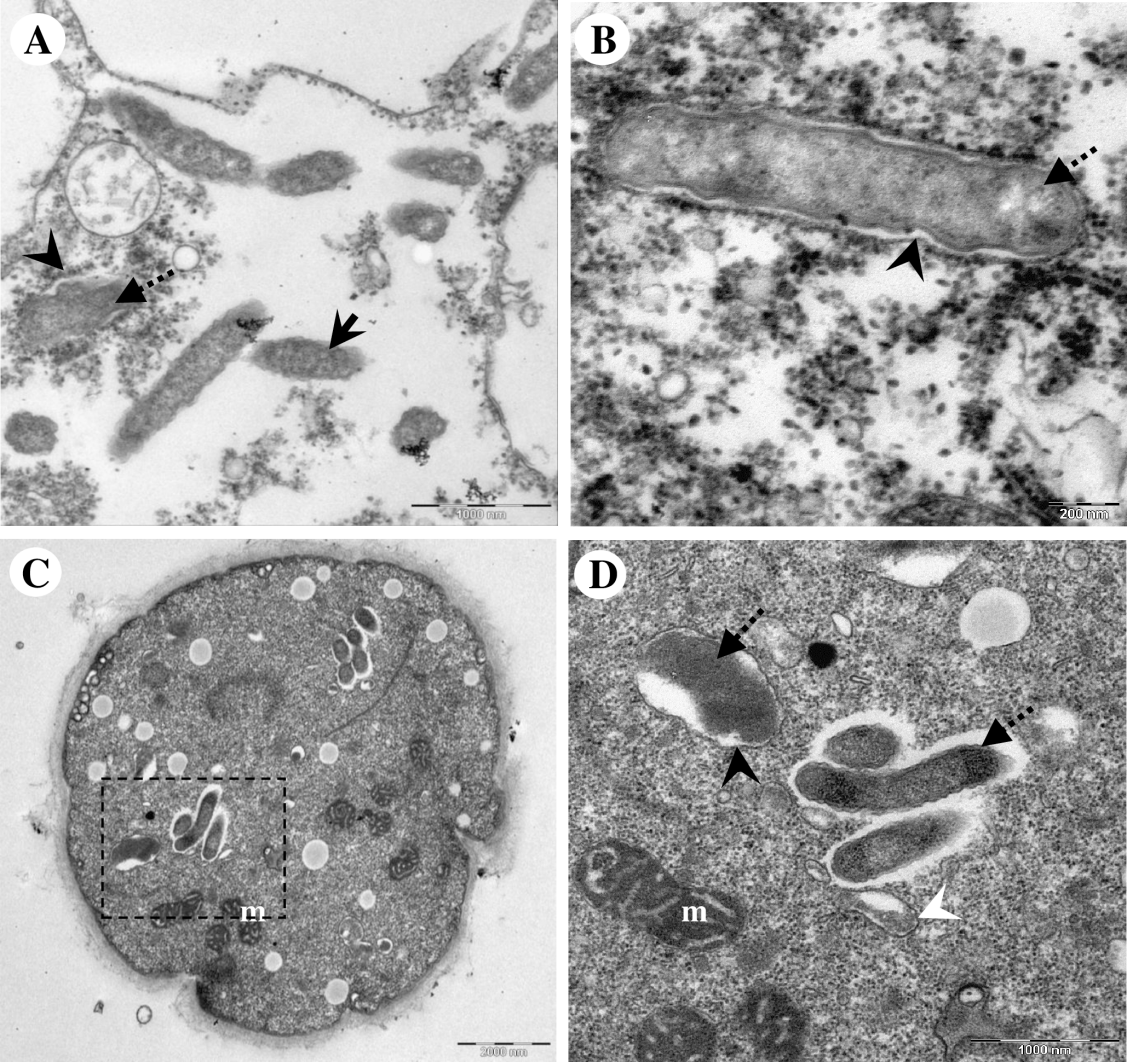
Transmission electron microscopic images demonstrating unknown membranous structure which selectively surrounded the morphologically altered endocytobionts. (A) Early form of membranous structure appending to a morphologically altered endocytobiont. Note the presence of intact endocytobionts in the surrounding. (B) Higher magnification showing a formed membranous vacuole which contained a morphologically altered endocytobiont. (C) Unknown membraneous vacuole observed inside a cyst of *Acanthamoeba* sp. HTH136. (D) Higher magnification of the square with dotted-line in (C). Note the membrane-bound endocytobiont appears disintegrated, and the vacuolar membrane is not entirely closed. Nearby are three endocytobiotic bacteria; the surface of these cells appeared slightly rough, however, they were not encircled by vacuole. An autophagic isolation membrane which engulfed cytoplasmic cargo is seen. Indicators = unknown membranous structure: ‘black arrow-head’; morphologically altered endocytobionts: ‘dotted-arrows’, and morphologically intact endocytobionts: ‘solid-arrows’, autophagic isolation membrane: ‘white arrow-head’, and mitochondria: m.

We compared the ultrastructures of the current unknown vacuoles to those of the autophagic and phagocytic vacuoles. A typical autophagic process was observed in *Acanthamoeba* cells; the autophagic isolation membrane first appended closely to the targeted mitochondria, this followed by the elongation of the isolation membrane to enclose the entire mitochondria, and eventually forming the double-membranous autophagosome (Fig 5). In regards to the current endocytobionts, isolation membrane-like structure was observed appending to the morphologically altered cells (Fig 6). In one instance, the entrapped endocytobiont appeared disintegrated, and granular materials were spotted draining into host cytoplasm via the opening at the vacuolar membrane (Fig 6).

**Fig 7:**
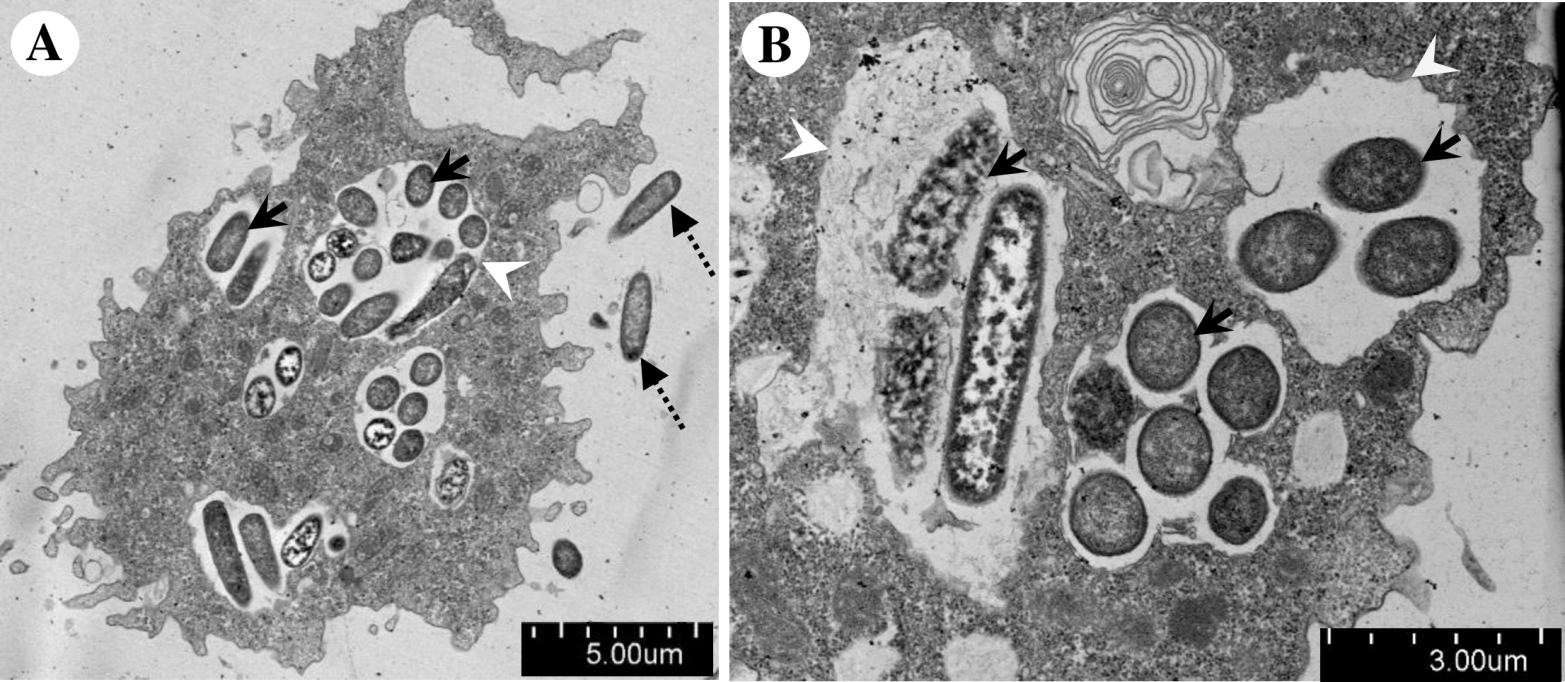
Transmission electron microscopic images illustrating the phagocytic vacuoles which were detected inside a trophozoite of *Acanthamoeba* sp. IMU4. (A) The amoeba was fed with *E*. *coli*. Numerous phagocytic vacuoles were observed in the cytoplasm. (B) Higher magnification illustrating different developmental stages of phagocytic vacuoles; some engulfed *E*. *coli* appeared intact whereas others appeared disintegrated. Indicators = phagocytic vacuoles: ‘arrow-heads’, free *E*. *coli*: ‘dotted-arrows’, and ingested *E*. *coli*: ‘solid-arrows’.

We then compared the current unknown vacuoles with phagocytic vacuoles. We cultured the endocytobiont-free IMU4 isolate with *E*. *coli* as food source and examined the cells by TEM. Variable sizes of phagocytic vacuoles were observed in the vegetative trophozoites, some phagocytic vacuoles were large enough to encircle more than ten ingested bacteria (Fig 7). A clear phagosomal membrane could be seen surrounding the ingested bacteria. Phagosome, the early phagocytic vacuole, was recognized by double-membranous vacuolar structure which contained engulfed, undigested *E*. *coli* (Fig 7). Phagolysosome, the late phagocytic vacuole, was recognized by the appearance of single-membranous vacuolar structure and the presence of partially degraded *E*. *coli* or their remnants (Fig 7). We noted there was a lack of similarity between the fine structure of the current unknown membranous vacuole to those of the phagocytic vacuole.

**Fig 8:**
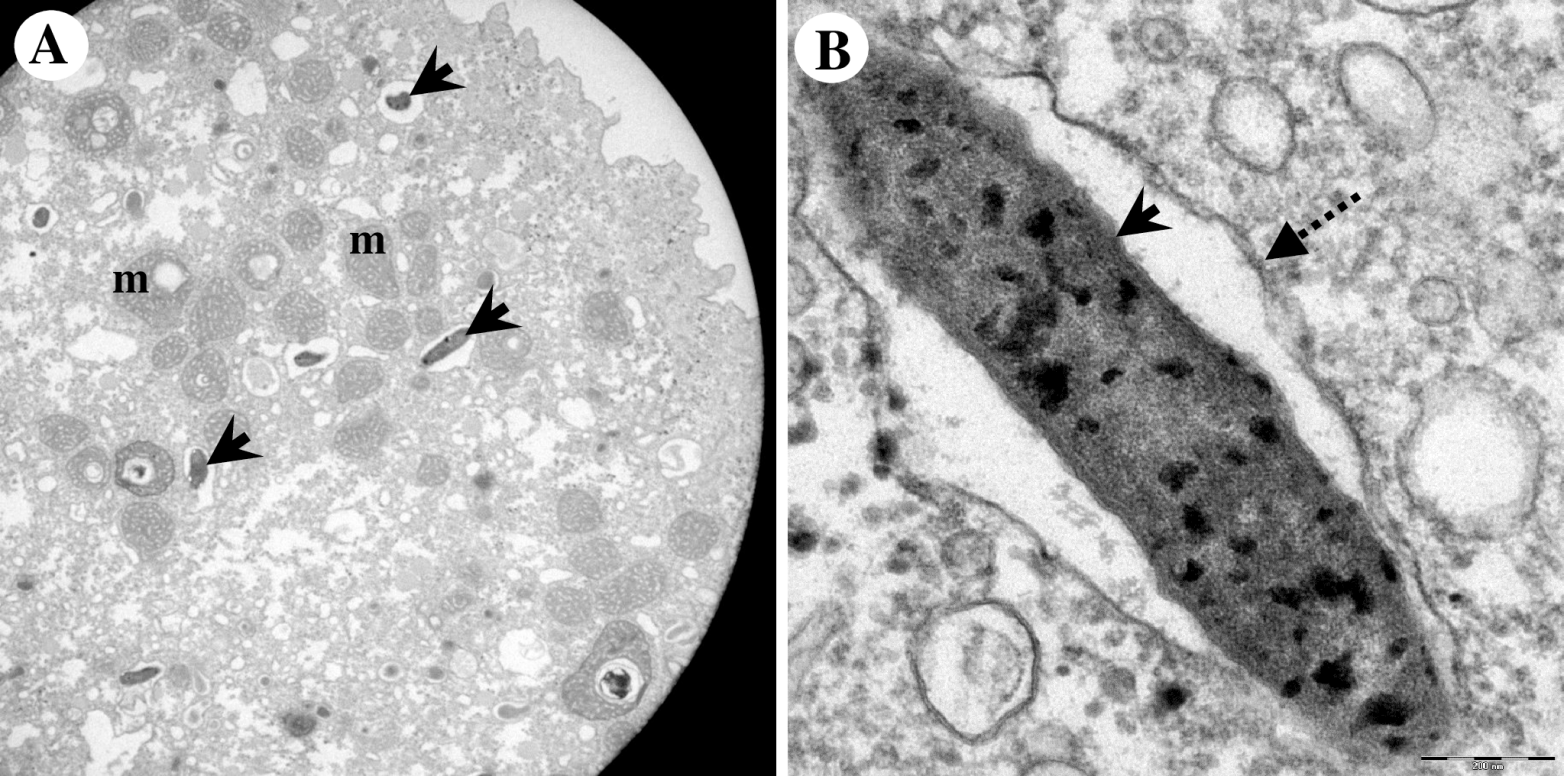
Morphological appearance of endocytobiont Endo_IMU7 inside *Acanthamoeba* sp. IMU7. (A) Overview of a trophozoite. The endocytobionts distributed randomly in cytoplasm of host. (B) Higher magnification of an endocytobiont. Note the cell was encircled by vacuolar membrane and the cell surface was coated with multiple electron-dense granules. Indicators = Endo_IMU7: ‘solid-arrows’, vacuolar membrane: ‘dotted-arrows’, and mitochondria: m.

**Fig 9:**
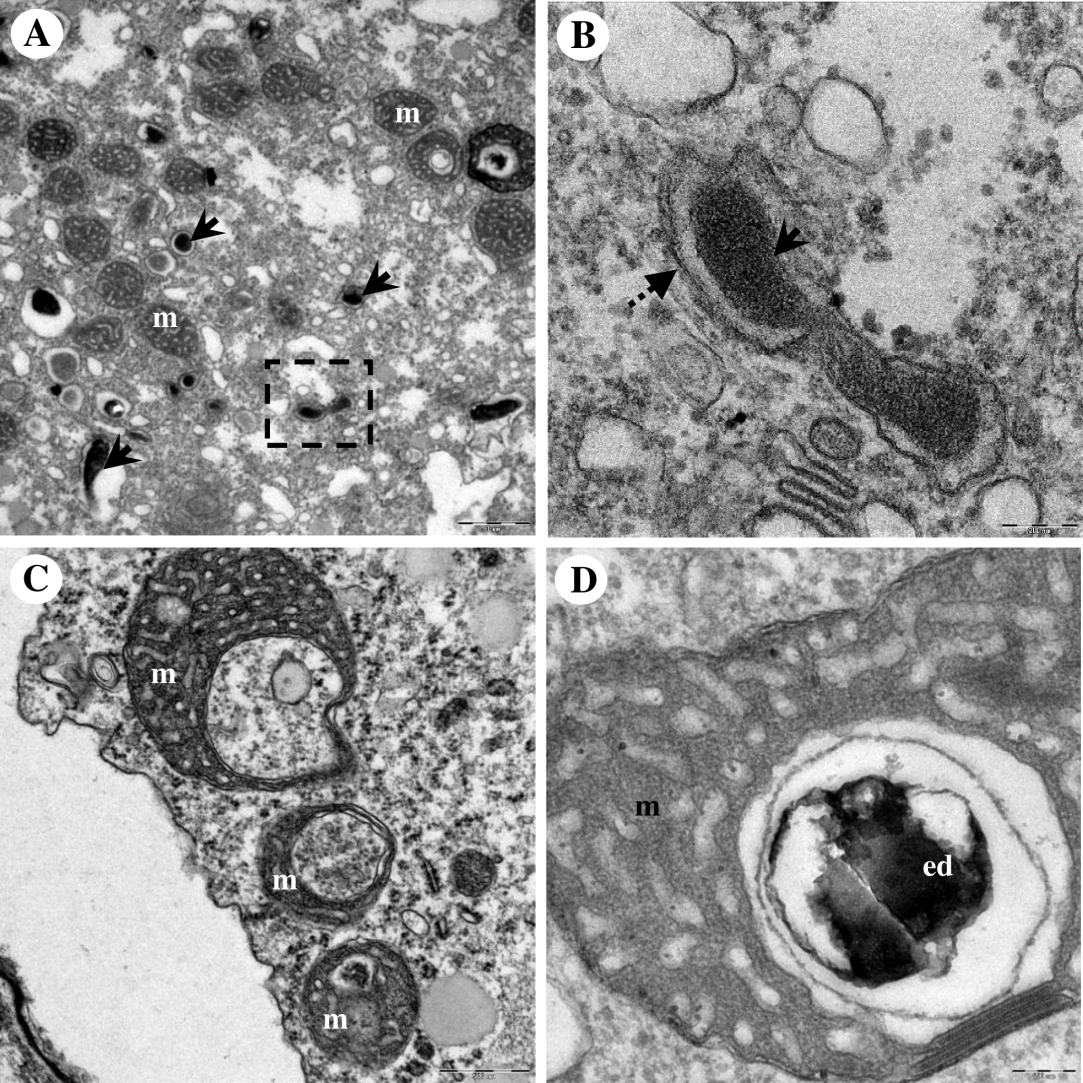
Transmission electron microscopy of *Acanthamoeba* sp. IMU7 bearing its endocytobiotic bacteria Endo_IMU7. (A) Transmission electron microscopic image showing a dividing endocytobiont (square with a dotted-line). (B) Higher magnification of the square with a dotted-line shows the cell replicates by binary fission. Note the concurrent bacterial and vacuolar membrane divisions. (C) TEM image showing the host’ mitochondria which appeared enlarged, vacuolated or with deposits accumulation. (D) Higher magnification of a morphologically altered mitochondrion. Note this organelle was filled with electron-dense deposit. Abnormal looking mitochondria were seen in the intact trophozoites. Indicators = Endo_IMU7: ‘solid-arrows’, vacuolar membrane: ‘dotted-arrows’, electron-dense granules: ed, and mitochondria: m.

**Fig 10:**
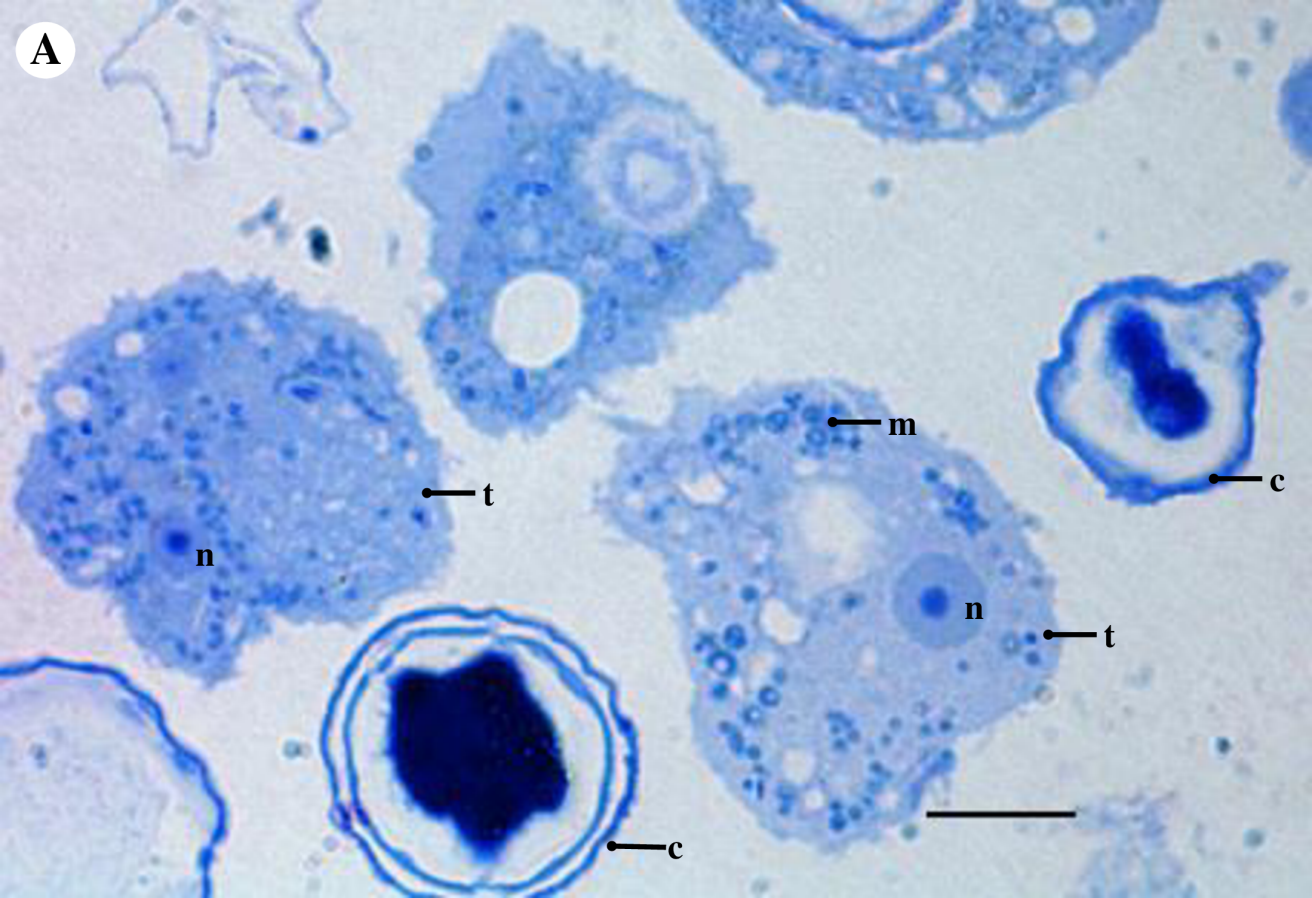
Light microscopic image on a toluidine-blue stained, semi-thin section of *Acanthamoeba* sp. IMU7 cells. Abnormal looking mitochondria were seen in the intact trophozoites. Indicators = cyst: c, mitochondria: m, nucleus: n, and trophozoite: t. Bar represents 10 μm.

**Fig 11:**
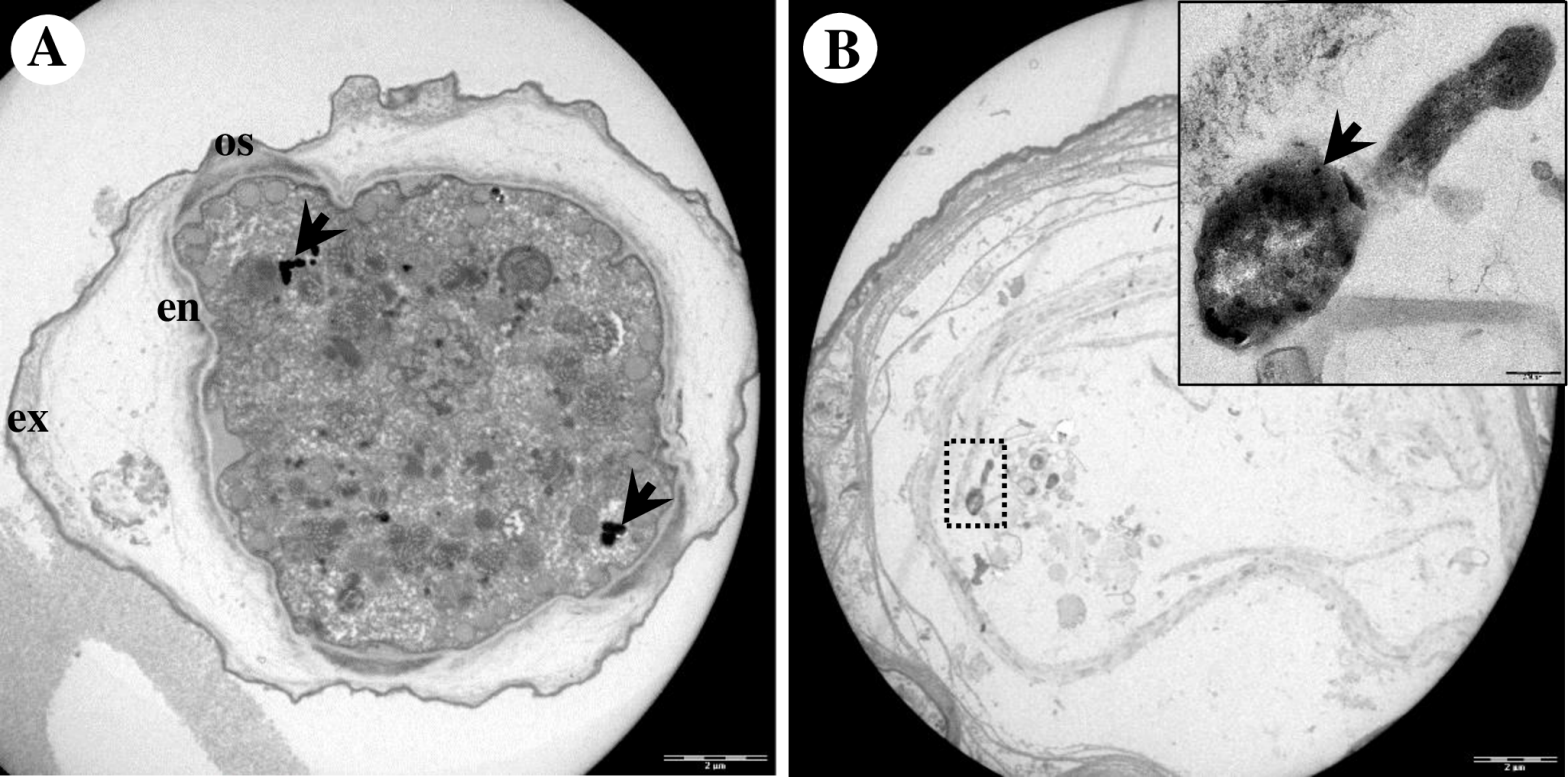
Ultrastructural appearance of Endo_IMU7 in cystic stage of amoebae host. (A) Cyst has relatively fewer endocytobionts compares to trophozoite. (B) An empty cyst containing a morphologically intact Endo_IMU7. Inset shows the enlarged image of the endocytobiont. Indicators: Endo_IMU7: ‘arrows’, endocyst: en, exocyst: ex, and ostiole: os.

Ultrastructural analysis by TEM showed that endocytobiont Endo_IMU7 was rod-shaped, distributed randomly throughout the host cytoplasm and was enclosed in vacuole. Multiple patches of electron-dense granules were noted on the cell surface (Fig 8). We noted each vacuole was occupied by a bacterium, possibly due to the concurrent bacterial and vacuolar membrane division (Fig 9). It was intriguing to observe mitochondria of host cells demonstrating various structural alternations such as enlargement, vacuolation and accumulation of dense deposits (Fig 9). We examined the toluidine-blue stained, semi-thin sections of host cells under a light microscope, and observed similar abnormal looking mitochondria in intact trophozoites (Fig 10). In cystic stage of host, Endo_IMU7 were fewer compared to the trophic stage of hosts (Fig 11).

## Discussion

*Acanthamoeba* sp. harbouring bacterial endocytobionts are commonly found in various natural soil and freshwater environments as well as in man-made structures such as the cooling towers, ventilation systems and water supply facilities [45–46]. FISH with bacterial oligonucleotide probes are widely used method in the detection and localization of bacterial endocytobionts in amoebae hosts [46–50]. Often, these probes are designed to hybridize to the bacterial 16S rRNA, and the molecules are divided into two categories based on their binding patterns. One category comprises of oligonucleotides which bind to the highly conserved region of the 16S rRNA; these probes are recognized as bacterial-domain specific owing to their ability to detect a wide range of bacteria [45, 47]. Another category of oligonucleotides bind to the variable region of the 16S rRNA sequences which is unique to the targeted bacteria; hence, these probes are only able to detect a specific species or group of bacteria [48–50].

Although FISH is an efficient tool for the detection of bacterial endocytobionts, the method is technically demanding [28]. Hence, in this study, we used a set of broad-range primer pairs which target the bacterial 16S rRNA genes for the detection of bacterial endocytobionts in our *Acanthamoeba* isolates [29]. When we performed PCR using this primer set, amplicons with the expected size were produced from all the current *Acanthamoeba* isolates. However, sequencing and Blastn analysis indicated only six amplicons matched closely to the bacterial 16S rRNA genes whereas the remaining eight matched to mitochondrial DNA sequences of different *Acanthamoeba* sp. We used the Primer-BLAST tool to check for primer mismatch and found nine nucleotides at the 3’-terminal of our forward primer and almost 95% of the reverse primer nucleotides to be complimentary to the sequences of various *Acanthamoeba* mitochondrial rRNA genes [51]. Presumably, in the absence of bacterial DNA, the primers primed to *Acanthamoeba* mitochondrial DNA and produced false-positive amplification. In fact, subsequent cell staining with Wright Giemsa solution and light microscopic examination confirmed the above eight investigated *Acanthamoeba* isolates to be endocytobiont-free (S2 and S3 Figs). Our observation highlighted the need of performing bacterial primer checking against the *Acanthamoeba* genome prior to their use in PCR screening for bacterial endocytobionts in the amoebae.

Our molecular analysis indicated Endo_11, Endo_IMU12, Endo_IMU13, Endo_IMU19 and Endo_HTH136 were affiliated to the previously described “*Ca*. Caedibacter acanthamoebae” whereas Endo_IMU7 was affiliated to the endosymbiont of *Acanthamoeba* UWC8 or the “*Ca*. Jidaibacter acanthamoeba” [8, 30, 32]. Indeed, our FISH results further supported such affiliations. “*Ca*. Caedibacter acanthamoebae” is a *Rickettsiales* bacteria of the *Holosporaceae* family [8, 52]. The phylogeny of *Alphaproteobacteria* class has been revised recently, as a result, *Holosporaceae* has been proposed to be promoted to order rank, the *Holosporales* ord. nov. [53–54]. Recently, the taxonomy of “*Ca*. Caedibacter acanthamoebae” has been further revised in a study which revisit the phylogeny of *Holosporales* bacteria. Consequent to the phylogenetic reconstruction, *Ca*. Caedibacter acanthamoebae” has been proposed to be transferred to a new genus “*Ca*. Paracaedimonas” gen. nov. and renamed as the “*Ca*. Paracaedimonas acanthamoeba” [31]. The endosymbionts of *Acanthamoeba* sp. UWC8 and UWC36 are *Rickettsiales* bacteria belong to the *Candidatus* Midichloriaceae family of the *Alphaproteobacteria* class [30, 55]. Lately, the endosymbiont of *Acanthamoeba* UWC36 has been characterized in detailed and renamed as “*Ca*. Jidaibacter acanthamoeba” [32]. Comparison of near-full-length 16S rRNA gene sequences indicated both *Rickettsiales* bacteria to have a 99.6% sequence similarity, however, at genomic level, they are quite different [30]. The genome size of the endosymbiont of *Acanthamoeba* sp. UWC8 is~1.6 Mb, having a small genome size which is typical to most *Rickettsiales* bacteria [55]. It is believed that *Rickettsiales* bacteria had undergone genome reduction through evolution, consequent to their adaptation as intracellular symbionts [56]. In contrast, “*Ca*. Jidaibacter acanthamoeba” has a large genome size of ~2.4 Mb due to the possession of genes similar to those which are present in the ancient counterparts, suggesting it has not undergone significant genome reduction as compared to other typical *Riskettsiales* bacteria, i.e. the endosymbiont of *Acanthamoeba* sp. UWC8 [32].

The overlapping positive fluorescence signals with the combined use of an endosymbiont-specific and a bacterial domain-specific probes in present FISH assays indicated single endocytobiont strain occupying each host isolate. However, we are aware that the bacterial domain-specific probe may not be entirely universal, hence, it may fail to detect the rare, less abundant, slow growing endocytobionts which may be present in our *Acanthamoeba* isolates. Likewise, our PCR assay with universal bacterial primers may encounter similar technical limitation. In regards to the molecular identification of bacterial endocytobionts, 16S rRNA gene high-throughput sequencing offers an ideal solution for determining heterogeneity of endocytobiotic bacteria hosted by a particular amoebae [57–58].

As the ultrastructural characters of “*Ca*. Caedibacter acanthamoebae”/“*Ca*. Paracaedimonas acanthamoeba”, the endosymbiont of *Acanthamoeba* UWC8 and “*Ca*. Jidaibacter acanthamoeba” are insufficiently explored to date, we used TEM to examine our isolates which are affiliated to these bacteria. Good-quality images revealed important information on the occurrence and association between the current endocytobionts and their hosts. Furthermore, we observed some of the Endo_12, Endo_19 and Endo_HTH136 cells to have different morphologies such as thickening of cell walls and cell surface roughness. As similar cellular features could be seen on dying or dead bacterial cells post-antimicrobial treatments, it is reasonable to believe these morphologically altered endocytobionts were unhealthy or non-viable [59–60]. We further noticed only the morphologically altered endocytobionts to be exclusively enclosed within these membranous vacuoles which were structurally similar to the autophagic vacuoles.

Autophagy is a lysozyme-associated degrading system which is conserved in eukaryotic cells. Autophagy plays various roles which are crucial for the survival of cells. Among the functions are removal of damaged organelles, eliminate incorrectly synthesized macromolecules, break down cellular structures for recycling, and protect eukaryotic cells from invading microbes [61–62]. Recently, the possible involvement of autophagy in regulating the intracellular localization of endosymbiont was described for “*Ca*. Fokinia solitaria” which was identified in the *Paramecium* Rio ETE_ALG 3VII strain. It was observed that the endosymbionts preferentially harboured at the host cortex and were non-membrane bound. However, those which occupied the inner part of cytoplasm were enclosed in autolysosomes. The authors suggested that “*Ca*. Fokinia solitaria” located in the inner part of the cytoplasm would be treated as pathogen by the host hence leading to their clearance by autophagy [50]. In *Acanthamoeba*, no similar observation has been reported although the role of autophagy has been well implicated in regulating amoebal cellular composition during encystation [63–64]. To the best of our knowledge, our TEM study is the first to illustrate the possible involvement of an autophagy-like process in regulating endocytobiont clearance in *Acanthamoeba* sp. We speculate that the persistent occurrence of defective endocytobionts can have a harmful effect, thus leading to their clearance by the amoebae hosts.

At present, we are unclear how this autophagic-like process could be initiated and regulated at the molecular level. However, we suggest the process is selective in nature and it is associated with the structural integrity of endocytobionts. In regard to mitochondria, an organelle which is known to be bacterial-derived endosymbiont of the ancient eukaryotic cells, the mitochondrial kinase PINK1 was shown to be involved in the selective removal of dysfunctional mitochondria. In healthy mitochondria, PINK1 kinase undergo rapid translocation and are broken down by protease at the inner mitochondrial membrane. However, when mitochondria became defective, the membrane potential would be altered, blocking the translocation of PINK1, leading to the accumulation of the kinase at the mitochondrial outer membrane. Such accumulation would attract and activate the ubiquitin-protein system, which then linked a series of host receptors and adaptor proteins to engage the isolation membrane to the defective mitochondria [65]. Finding similar membrane-associated regulators which play roles in sensing and assisting in the selective removal of defective endocytobionts in *Acanthamoeba* sp. will be the direction for future study.

As for the Endo_IMU7, the presence of malformed host mitochondria strongly suggested their occurrence may have a negative impact on the host’s wellbeing. In addition, the low number of Endo_IMU7 in *Acanthamoeba* cysts indicated that the intracellular bacteria may have a poor adaptation to the encystation process. Hence, it is intriguing to know how prolonged a biotic relationship can be maintained between these two microorganisms. A study to track the bacterial and host cellular changes during encystation should shed some light on their interactions.

## Supporting information

S1 Fig **Double FISH images of (A and B) Endo_IMU12 and (C and D) Endo_IMU19.** The probes used in this analysis were: (A and C) FITC-labelled probe specific to “*Ca*. Caedibacter acanthamoebae”/“*Ca*. Paracaedimonas acanthamoeba”, (B and D) Cy3-labelled oligonucleotide bacterial-domain specific probe S-D-Bact-0338-a-A-18. For each combination of probes, an identical microscopic field was visualized by a fluorescence microscope. Bars represent 10 μm.
(TIF)

S2 Fig Light microscopic examination on Wright Giemsa stained *Acanthamoeba* sp. IMU12 revealing the presence of bacterial endocytobionts. Indicators = Rod-shape, purplish pink bacteria endocytobionts: ‘black arrows’, nucleus: n, and cytoplasm: cy. Bar represents 10 μm.
(TIF)

S3 Fig Light microscopic examination on Wright Giemsa stained *Acanthamoeba* sp. IMU4 revealing the absence of bacterial endocytobiont. Indicators = nucleus: n, and cytoplasm: cy Bar represents 10 μm.
(TIF)
