# Supplementary figures and images for: Identification and ultrastructural characterization of Acanthamoeba bacterial endocytobionts belonging to the Alphaproteobacteria class
Source: PLoS One. 2018 Oct 24;13(10):e0204732. doi: 10.1371/journal.pone.0204732 (PMC6200196; doi:10.1371/journal.pone.0204732)

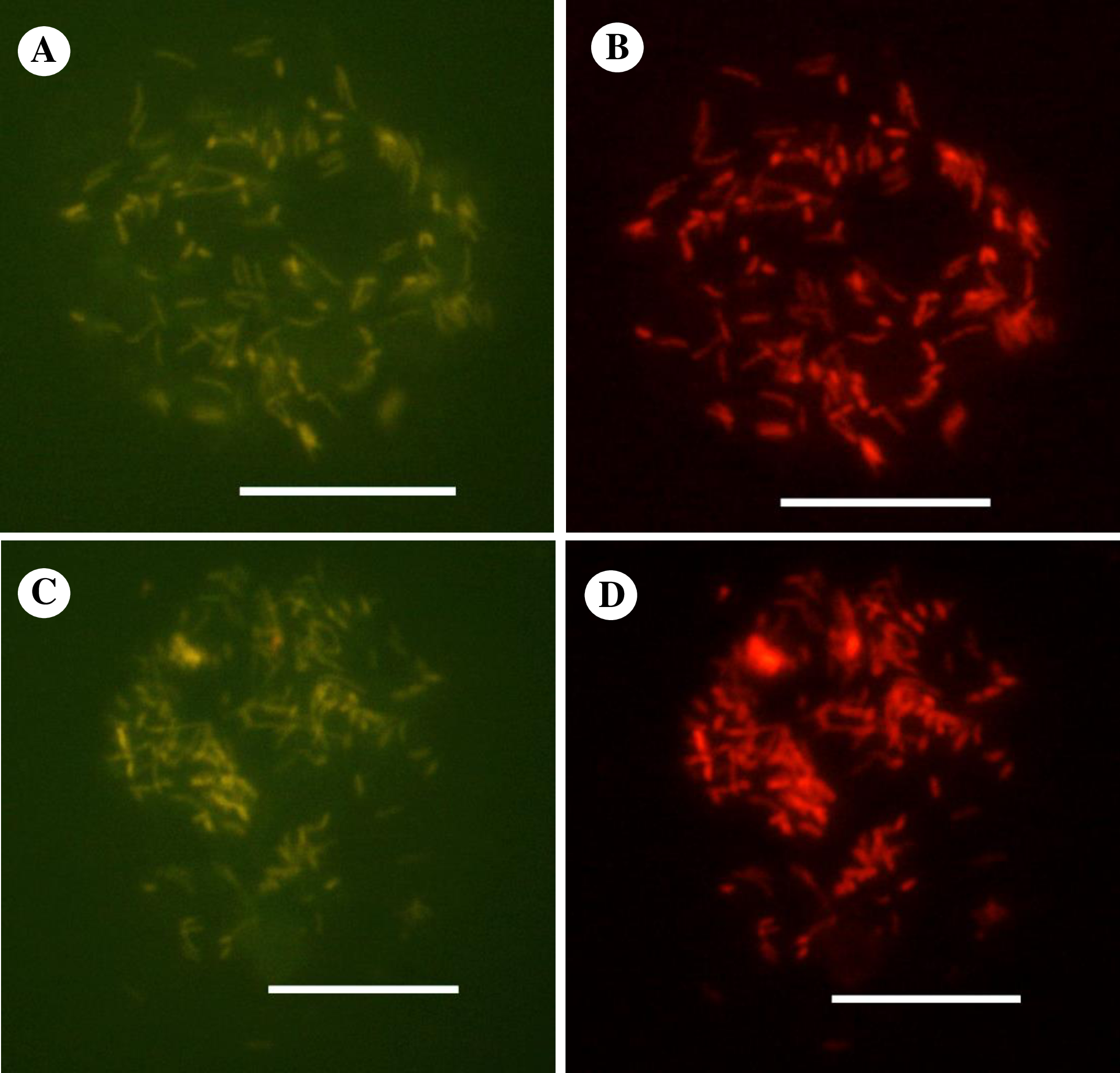

Supplement: S1 Fig — Double FISH images of (A and B) Endo_IMU12 and (C and D) Endo_IMU19. The probes used in this analysis were: (A and C) FITC-labelled probe specific to “Ca. Caedibacter acanthamoebae”/“Ca. Paracaedimonas acanthamoeba”, (B and D) Cy3-labelled oligonucleotide bacterial-domain specific probe S-D-Bact-0338-a-A-18. For each combination of probes, an identical microscopic field was visualized by a fluorescence microscope. Bars represent 10 μm. (TIF) [file pone.0204732.s001.tif]

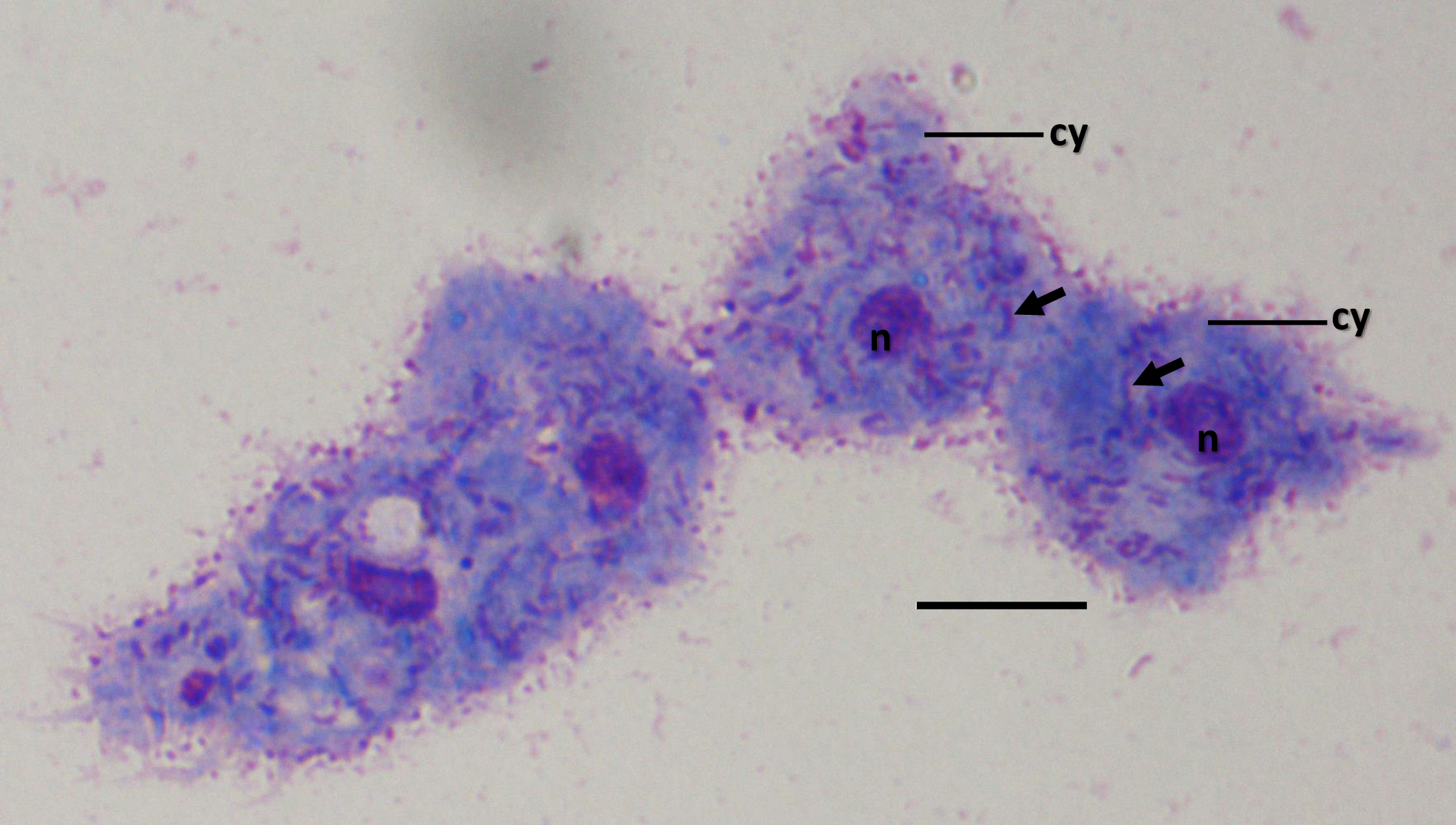

Supplement: S2 Fig — Indicators = Rod-shape, purplish pink bacteria endocytobionts: ‘black arrows’, nucleus: n, and cytoplasm: cy. Bar represents 10 μm. (TIF) [file pone.0204732.s002.tif]

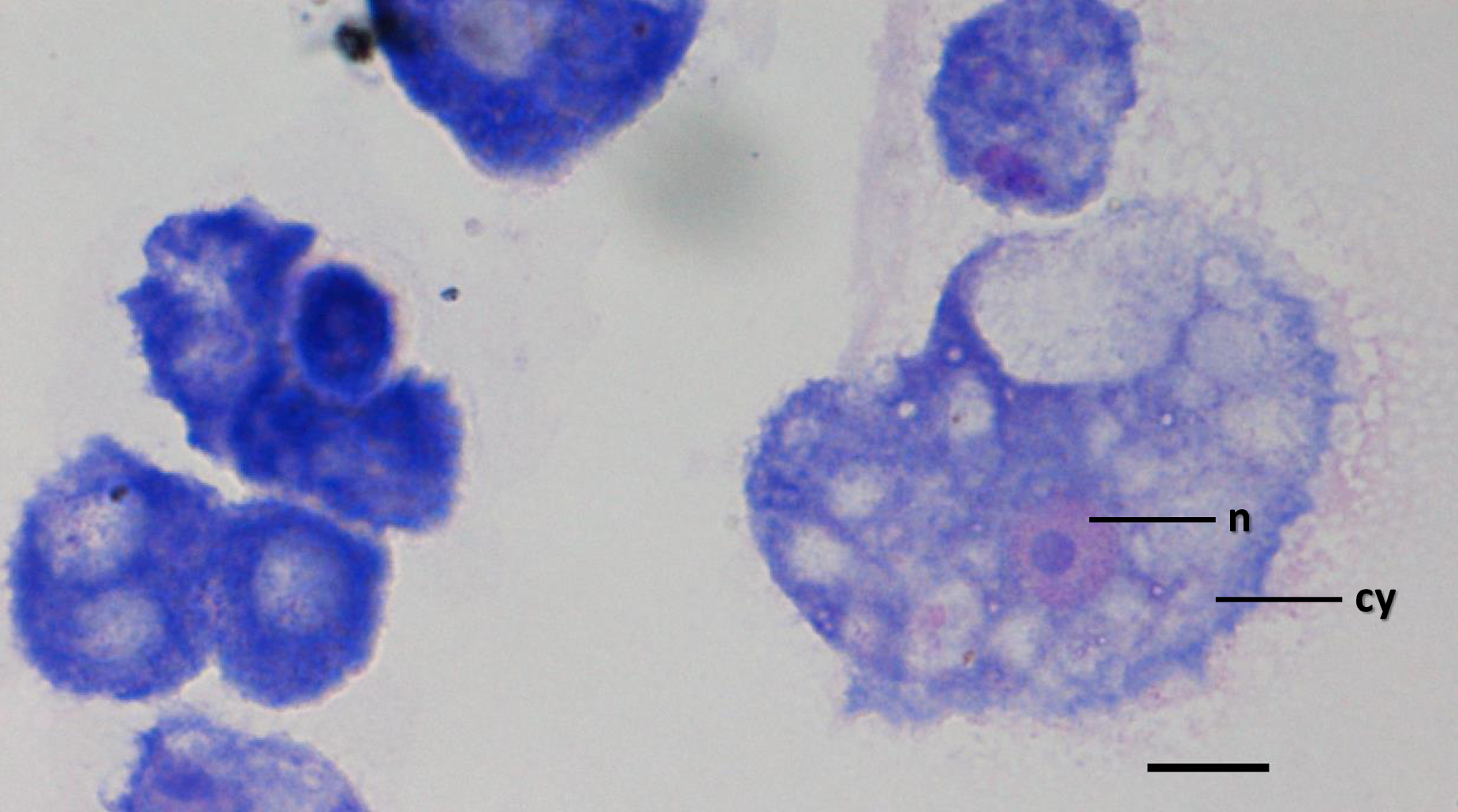

Supplement: S3 Fig — Indicators = nucleus: n, and cytoplasm: cy Bar represents 10 μm. (TIF) [file pone.0204732.s003.tif]
